# Histologic Characterization of Tumor-Adjacent Mammary Adipose Tissue in Normal-Weight and Overweight/Obese Patients with Triple-Negative Breast Cancer

**DOI:** 10.3390/cancers16203515

**Published:** 2024-10-17

**Authors:** Marietta Wolf, Christoph Brochhausen, Vignesh Ramakrishnan, Sabine Iberl, Jonas Roth, Stephan Seitz, Ralph Burkhardt, Sonja C. Stadler

**Affiliations:** 1 Institute of Clinical Chemistry and Laboratory Medicine, University Hospital Regensburg, 93053 Regensburg, Germanyralph.burkhardt@klinik.uni-regensburg.de (R.B.); 2 Department of Operative Dentistry and Periodontology, Center for Dental Medicine, Medical Center—University of Freiburg, Faculty of Medicine, University of Freiburg, 79106 Freiburg im Breisgau, Germany; 3 Institute of Pathology, Medical Faculty Mannheim, University Heidelberg, 69120 Mannheim, Germany; 4 Institute of Pathology, Regensburg University, 93053 Regensburg, Germany; 5 Department of Gynecology and Obstetrics, University Medical Centre Regensburg, 93053 Regensburg, Germany

**Keywords:** triple-negative breast cancer, obesity, adipose tissue, lipid metabolism, tumor microenvironment, BMI

## Abstract

**Background:** Obesity is a risk factor of several types of cancer, including breast cancer. In this study, we aimed to histologically characterize the adipose tissue of the tumor microenvironment (TME) of triple-negative breast cancer (TNBC) in overweight/obese versus normal-weight patients. **Methods:** TNBC tissue sections from normal-weight (BMI_<25_) and overweight/obese patients (BMI_≥25_) were stained with antibodies against CD68, CD163, CD31, CD34, and vimentin. At the invasive tumor front, positive cells were counted in tumor adjacent adipose tissue (AT) and within cancer tissue (CT). Further, the size of the tumor-adjacent and distant mammary adipocytes was determined in perilipin stained sections. Expression of ANGPTL4, CD36 and FABP4, proteins involved in fatty acid metabolism, was analyzed in marginal tumor cells using an immune reactive score. **Results:** Overweight/obese TNBC patients had significantly larger adipocytes, higher numbers of CD163+ macrophages (BMI_<25_: 2.80 vs. BMI_≥25_: 10.45; *p* = 0.011) and lower numbers of CD31+ (BMI_<25_: 4.20 vs. BMI_≥25_: 2.40; *p* = 0.018) and CD34+ (BMI_<25_: 14.60 vs. BMI_≥25_: 5.20; *p* = 0.045) cells as markers of angiogenesis in the AT as well as a higher frequency of cancer-associated-fibroblast-like cells in the AT and CT (BMI_<25_: 7.60 vs. BMI_≥25_: 25.39 in total; *p* = 0.001). Moreover, expression of CD36 (BMI_<25_: 2.15 vs. BMI_≥25_: 2.60; *p* = 0.041) and ANGPTL4 (BMI_<25_: 6.00 vs. BMI_≥25_: 9.80; *p* = 0.026) was elevated in the TNBC cells of overweight/obese patients. **Conclusions:** Our data suggest BMI-related changes in the TME of overweight/obese TNBC patients, including hypertrophied adipocytes, reduced vascularization, more M2-like macrophages and CAF-like cells, and an increase in the expression of fatty acid metabolizing proteins in marginal tumor cells, all contributing to a more tumor-promoting, immunosuppressive environment.

## Simple Summary

Obesity increases the risk of several cancers, including breast cancer. With global obesity rates rising, it is important to understand how excess fat accumulation influences cancer development and progression. This study examined how overweight/obesity affects the breast adipose tissue around triple-negative breast cancer (TNBC), a particularly aggressive type of breast cancer. Therefore, tissue samples from overweight/obese and normal weight patients with TNBC were compared. We found that overweight/obese TNBC patients had larger fat cells, more immune cells that could support cancer growth, and fewer cells related to new blood vessel formation in the tumor-surrounding environment. Overweight/obese patients also showed higher numbers of special fibroblast cells with potential tumor-promoting functions. Moreover, tumor cells at the invasive front, which are in close contact with fat cells, showed higher levels of proteins that metabolize fatty acids, suggesting altered lipid handling in cancer cells in overweight/obese TNBC patients. Together, these findings imply that obesity is associated with distinct changes in the tumor-adjacent adipose tissue in TNBC, potentially affecting cancer aggressiveness. Understanding these changes may help to develop more effective treatment strategies for obese TNBC patients, which may improve their prognosis and overall health outcomes.

## 1. Introduction

Breast cancer is the most prevalent malignant tumor and a leading cause of cancer-related deaths in women worldwide [1]. Established risk factors for breast cancer include age, genetic predisposition, pregnancies, and lifestyle factors such as alcohol consumption and smoking [2]. More recently, overweight and obesity have also been recognized as risk factors for breast cancer development and progression. Epidemiological studies have consistently linked obesity and excess accumulation of adipose tissue to an elevated risk of breast cancer and poorer prognosis [2,3,4]. This not only affects the more prevalent hormone receptor-positive tumors, but also triple-negative (TN) tumors, where targeted treatment options are still very limited [5]. With globally rising incidences of overweight and obesity, understanding the details of the interaction between excessive adipose tissue and breast cancer is an important task.

The stroma of breast tumors is mainly composed of adipose tissue, comprising various cell types and extracellular matrix components, which collectively shape the tumor microenvironment (TME). Most studies investigating the association of obesity and breast cancer focused on the systemic impact of excess adipose tissue. Less attention has been paid to local alterations within the mammary adipose tissue microenvironment (ATME) that potentially contribute to cancer development and progression in obese patients [6]. This microenvironment, characterized by diverse cell populations including adipocytes, fibroblasts, immune and endothelial cells and extracellular matrix molecules, plays a pivotal role in tumor initiation and progression by delivering tumor-promoting molecules [6].

In obesity, adipocytes become hypertrophic and store elevated amounts of triacylglycerols (TAGs) along with higher secretion of steroid hormones, adipokines and pro-inflammatory cytokines such as TNFα, IL-1β, IL-6, and PAI-1 [6,7]. These molecules attract immune cells, promoting a state of chronic low-grade inflammation, which fosters breast tumor initiation and progression [8]. Additionally, hypertrophic adipocytes become stressed and eventually die, triggering the infiltration and activation of phagocytic macrophages [6,9]. These macrophages form crown-like structures (CLSs) to scavenge lipids and cellular debris. CLSs are considered biomarkers for adipose tissue inflammation and indicators of metabo-inflammation producing high levels of pro-inflammatory mediators [6,10]. In breast cancer, CLSs positively correlate with patient BMI and adipocyte size, and potentially indicate a worse clinical prognosis [11].

Obesity-associated adipose tissue inflammation along with an increase in tumor-associated macrophages within the mammary gland stroma may also enhance fibroblast activation and adipose tissue fibrosis [12]. Hyper-activated fibroblasts, termed cancer-associated fibroblasts (CAFs), contribute to cancer pathogenesis through the production of growth factors, cytokines, and chemokines, as well as matrix remodeling. They deposit matrix components such as fibrillar collagen and fibronectin, altering ECM stiffness [12,13,14]. The ECM alterations disrupt the biochemical and mechanical functions of mammary tissue, exacerbating the inflammatory impact of the ATME in obese individuals [6]. Additionally, obesity-induced vascular dysfunction and hypoxia in adipose tissue further contribute to a proinflammatory environment [8].

Moreover, adipocytes within the ATME exert profound effects on cancer cell metabolism [15]. Recent studies have demonstrated that breast cancer cells may induce lipolysis together with a phenotypic change in neighboring adipocytes [7]. These cells, termed cancer-associated adipocytes (CAAs), exhibit an altered morphology and secrete increased amounts of proteases and interleukins, promoting tumor aggressiveness [16]. Importantly, CAAs supply fatty acids to breast cancer cells: We and others have shown that breast cancer cells are able to take up fatty acids supplied by surrounding CAAs, which are stored in newly formed intracellular lipid droplets and/or used for fatty acid oxidation [17,18,19,20]. Important molecules involved in the lipolysis, transport, and uptake of exogenous fatty acids include the fatty acid binding protein 4 (FABP4), the fatty acid tissue translocase CD36, and the angiopoietin-like protein 4 (ANGPTL4), which have all been recently implicated in tumor biology [17,21,22,23].

Despite advances in understanding the link between obesity and breast cancer, the histological features of the ATME of triple-negative breast cancer, particularly in overweight and obese patients, have not been elucidated. In this study, we aimed to address this by histologically characterizing the ATME in TNBC patients, comparing overweight/obese individuals with normal-weight counterparts. Through detailed histological analysis utilizing specific markers for adipocytes, macrophages, endothelial cells, and fibroblasts, we describe significant alterations in the inflammatory and metabolic milieu of the TNBC ATME in overweight/obese patients.

## 2. Material and Methods

### 2.1. Study Design and Patient Material

Clinical data and archived formalin-fixed, paraffin embedded tissue blocks were obtained from a cohort of women who underwent partial or total mastectomy for treatment of triple-negative breast cancer (T1–T3; N0–N1; histological grades II and III) between 2011 and 2020 at the Department of Gynecology and Obstetrics, University Medical Center Regensburg. Patients selected for the study had been diagnosed with cancer for the first time and must not have received any form of neoadjuvant radio- or chemotherapy. Patients with chronic inflammatory diseases and auto-immune diseases were excluded from this study. In addition, FFPE samples had to fulfil the following criteria: (1) Presence of adipose tissue appropriate for analysis directly adjacent to the resected tumor. (2) Availability of a sample of adipose tissue with >2 cm distance from the tumor margin. In total, 30 cases were selected for this study and divided into two groups according to their body mass index (BMI): lean/normal weight (BMI_<25_, n = 10) and overweight/obese (BMI_≥25_; n = 20). The study was approved by the Ethics Committee of the University of Regensburg (reference number: 21-2314-14).

### 2.2. Immunohistochemistry

The antibodies and dilutions used for immunohistochemistry (IHC) are listed in Supplemental Table S1. The IHC stainings using antibodies against CD68 for macrophages, CD163 for M2-like polarized macrophages, vimentin for fibroblasts, and CD31 and CD34 for endothelial cells were conducted fully automatically with a BenchMark Ultra IHC automated staining instrument (Ventana Medical Systems, Roche Diagnostics, Mannheim, Germany) and staining protocols provided by the manufacturer. Stainings for ANGPTL4, CD36, FABP4, and perilipin were conducted manually. In brief, FFPE tissue sections were cut at a 2.5 µm thickness, deparaffinised in xylene, and rehydrated with four chambers of 100% and 70% alcohol and two chambers of water. Afterwards, the antigens were unmasked in a citrate buffer (pH 6) using the heat of a steamer followed by the blocking of free aldehyde groups in 50 mM glycine. Endogenous peroxidases were inhibited by treating the samples with 3% hydrogen peroxide. Slides were then incubated overnight in a humidity chamber with primary antibodies diluted in 0.5% BSA, followed by incubation with labeled secondary antibodies. The targeted antigens were detected with Bright-DAB and the nuclei were counterstained with hematoxylin. The slides were dehydrated with 70% and 100% alcohol and, lastly, xylene before coverslipping. All reactions were stopped by rinsing the tissues at least once with PBS (pH of 7.4)/0.3% Triton X/0.05% Tween 20 between each step. Staining specificity (negative control) was assessed by omitting the primary antibody incubation step. All manual stainings were conducted evenly across patient groups to minimize batch effects.

### 2.3. Analysis of IHC Stainings

All slides were scanned (Pannoramic 1000 whole-slide scanner, 3D-Histech) and evaluated with virtual microscopy software Case Viewer V2.2 (3D-Histech; 10 high-power fields at 40× magnification). If possible, the same high-power fields in consecutive slides of a given tissue sample were selected for analysis across the different antibody staining. For analysis, different sections of the high-power fields were used: CD36, ANGPTL4, and FABP4 stainings were evaluated within TNBC cells at the invasive front only. Thus, ANGPTL4, FABP4, and CD36 stainings were analyzed in high-power fields featuring two-thirds of the cancer tissue (invasive front) and one-third of the adjacent AT. All other stainings were evaluated within the tumor invasive front and adjacent adipose tissue. Therefore, CD31, CD34, CD68, CD163, perilipin, and vimentin stainings were analyzed in high-power fields featuring one-third cancer tissue (invasive front) and two-thirds AT.

### 2.4. Evaluation of Macrophages and CLS

To avoid double counting and potential confusion with other cell types, only CD68+ and CD163+ cells with a relevant size and a clear nucleus were analyzed. If the borders between macrophages were blurred, the nuclei were counted. In a subsequent step, crown-like macrophage structures around adipocytes were counted in the CD68 IHC stainings. CLSs were counted if at least two-thirds of the adipocyte were surrounded by macrophages.

### 2.5. Evaluation of Blood Vessels

For detection of angiogenesis, stainings were performed with CD31 and CD34 antibodies to compensate for the limitations in sensitivity and specificity of the single markers [24]. Only intensely colored cells were analyzed. Vessels running lengthways to the cutting direction were only counted once. We counted single-stained structures independently of size or lumen.

### 2.6. Evaluation of Fibroblasts

Vimentin was used to evaluate fibroblasts and cells with a CAF-like phenotype. Therefore, vimentin-positive cells were evaluated based on morphology taking into account cell size, nucleus size, and cell shape. Fibroblasts are generally described as spindle-shaped cells, with CAFs being significantly larger than normal fibroblasts featuring a large active, indented nucleus as well as elongated cytoplasmic processes [13].

### 2.7. Perilipin Staining and Quantification of Adipocyte Size

Perilipin (a marker protein surrounding lipid droplets) was used to assess adipocyte size. For each patient, the average size of the adipocytes was quantified at three different locations: (1) first-row adipocytes directly bordering the invasive front, (2) second-row adipocytes adjacent to the first-row adipocytes, and (3) peripheral adipocytes at a minimum distance of 2 cm from the invasive tumor margin. To quantify the average size, adipocytes in five representative HPFs of each area were circled manually with the software Omero (V5.6; https://www.openmicroscopy.org/omero/). Afterwards, the area of the adipocytes was calculated by an algorithm derived from a deep learning neural network based on U-Net architecture, which is trained to segment out adipocytes. The semantically segmented adipocytes were further isolated and measured using morphological algorithms with the help of a “regionprops” library [25].

### 2.8. Evaluation of Cancer Cells for Molecules Associated with Fatty Acid Metabolism

CD36, ANGPTL4 and FABP4 expression in cancer cells was analyzed by applying an immune reactive score (IRS): Firstly, the staining intensity was classified on a scale from 0 to 3 [0 = no staining, 1 = weak staining, 2 = medium staining, 3 = intense staining]. Secondly, the percentage of stained cancer cells was classified on a scale from 0 to 4 [0 = no cells stained, 1 = 1–10% cells stained, 2 = 11–50% cells stained, 3 = 51–80% cells stained, 4 = 81–100% cells stained]. If in doubt, the next higher classification level was selected in both steps. In a third step, the product of the two scores was calculated to derive an IRS. Endothelial cells, which may also stain CD36 positive, were not counted and thus excluded from the analysis.

### 2.9. Statistical Analysis

Statistical analysis was performed with GraphPad Prism 9. For any given patient and parameter, average values were derived across 10 high-power fields [in the case of perilipin, only 5 high-power fields]. Statistical testing was performed with a Mann–Whitney U test. A *p*-value < 0.05 was considered as statistically significant. Values are displayed as a median followed by the interquartile range (IQR, 25% and 75%) in brackets.

## 3. Results

### 3.1. Patient Characteristics

This study comprised 30 female patients undergoing partial or complete mastectomy as an initial treatment for first-time diagnosed triple-negative breast cancer (TNBC). The clinico-pathologic characteristics of the patients are shown in Table 1. The cohort was divided into two groups according to the BMI of the patients: (1) lean/normal (BMI_<25_, n = 10) with a median BMI of 22.40 and (2) overweight/obese (BMI_≥25_, n = 20) with a median BMI of 29.30.

### 3.2. Macrophage Infiltration and Crown-like Structures in Mammary Tumor and Adipose Tissue

**Figure 1:**
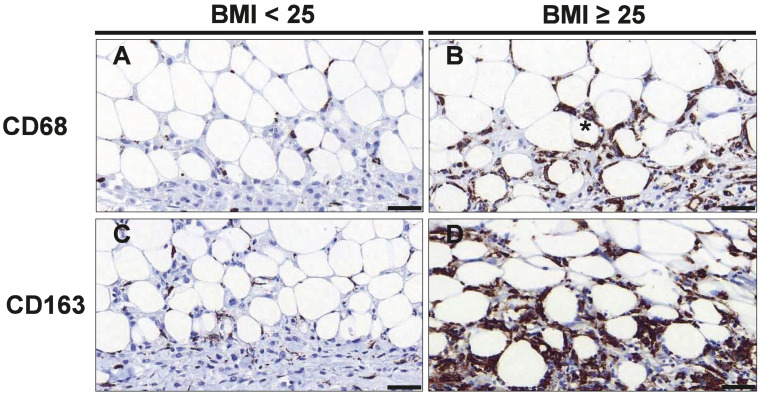
CD68+ and CD163+ macrophages are increased in tumor-adjacent adipose tissue of overweight/obese TNBC patients. Representative images of immunohistochemical staining with antibodies against CD68 (**A**,**B**) and CD163 (**C**,**D**) in normal weight (BMI<_25_) and overweight/obese patients (BMI_≥25_) with TNBC. The asterisk (*) denotes a typical crown-like structure (CLS), Scale bar 50 µm (magnification 40×).

We first investigated if the number and activation state of macrophages within the invasive tumor front and surrounding adipose tissue differed between normal and overweight/obese patients. Therefore, sections containing tumor and tumor-adjacent adipose tissue were stained with antibodies against CD68 and CD163 to detect M2-like polarized macrophages (Figure 1).

The numbers of both, CD68+ and CD163+ cells, were significantly increased in tumor-adjacent adipose tissue in overweight/obese patients (Figure 1A–D). In particular, overweight/obese patients displayed 3.73-fold (*p* = 0.011) more CD163+ M2-like macrophages in tumor-adjacent adipose tissue as compared to normal-weight patients, whereas the number of CD68+ cells was increased by 1.7-fold (*p* = 0.038) (Table 2). Likewise, overweight/obese patients showed a strong tendency for higher numbers of CD68+ (1.8-fold, *p* = 0.119) and CD163+ (3.80-fold; *p* = 0.062) cells in the tumor tissue. When analyzing the total number of cells in the tumor and tumor-adjacent adipose tissue together, only CD163+ cells were significantly more abundant in overweight/obese patient (4.06-fold; *p* = 0.039; Table 2). Thus, samples from overweight/obese patients were characterized by a distinct increase in CD163+ M2-like polarized macrophages, which was particularly pronounced in tumor-adjacent adipose tissue.

We next evaluated the abundance of crownlike structures as a marker of adipose tissue inflammation. Overweight/obese patients exhibited significantly more crown-like structures in tumor-adjacent adipose tissue than normal-weight patients (6.80-fold; *p* = 0.001; Figure 1A,B; Table 2).

### 3.3. Endothelial Cells and Angiogenesis

**Figure 2:**
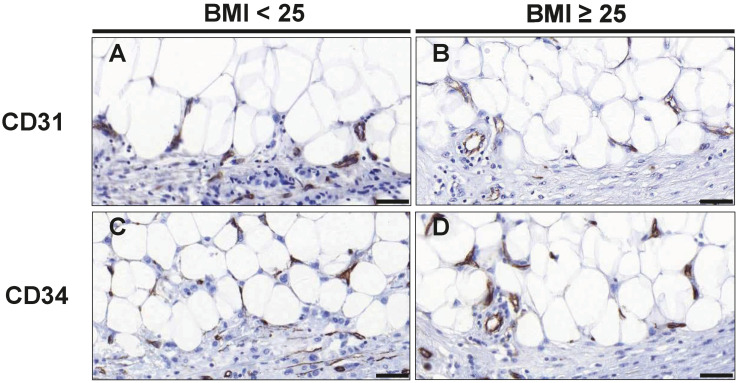
Staining of CD31+ and CD34+ endothelial cells to assess microvessel formation. Representative images of immunohistochemical staining with antibodies against the endothelial cell markers CD31 (**A**,**B**) and CD34 (**C**,**D**) in normal weight (BMI_<25_) and overweight/obese patients (BMI_≥25_) with TNBC. Scale bar 50 µm (magnification 40×).

To assess the effects of overweight/obesity on angiogenesis in tumor-adjacent adipose tissue, we used CD31 and CD34 as endothelial markers for microvessel formation. A combination of both markers was applied, as previous studies suggested that CD34 appears more sensitive, but less specific than CD31 for endothelium [26]. Notably, both, the number of CD31+ and CD34+ cells, was significantly lower in the tumor-adjacent adipose tissue of overweight/obese patients: While CD31+ cells were reduced by 43% (*p* = 0.018), CD34+ cells were reduced by 64% (*p* = 0.045) (Table 3 and Figure 2). We also assessed the number of CD31+ and CD34+ cells within the tumors. Overall, CD31+ and CD34+ cells were much more abundant within cancer tissue as compared to tumor-adjacent adipose tissue. However, the numbers of intratumoral CD31+ and CD34+ cells were not significantly different between normal-weight and overweight/obese patients (Table 3).

### 3.4. Fibroblast Distribution

**Figure 3:**
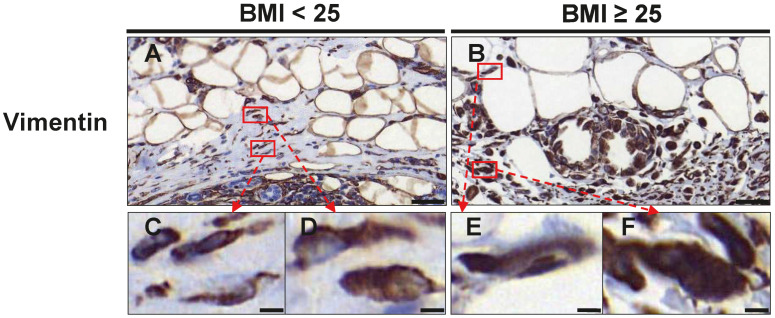
Assessment of fibroblasts and CAF-like cells at the invasive front and tumor-adjacent adipose tissue. Representative images of immunohistochemical staining with an antibody against vimentin in normal weight (BMI_<25_) (**A**) and overweight/obese patients (BMI_≥25_) (**B**) with TNBC. Scale Bar 50 µm. Lower panel (**C**–**F**) shows magnifications of the respective boxes in (**A**,**B**). Regular spindle-shaped fibroblasts are shown in (**C**,**E**). CAF-like cells with a larger cell size and nuclei are shown in (**D**,**F**). Scale bar 5 µm.

The distribution of fibroblasts at the invasive front and tumor-adjacent adipose tissue was analyzed by vimentin staining (Table 4, Figure 3). Cells with a typical spindle-shaped morphology were counted as regular fibroblasts, whereas larger vimentin positive cells with larger, indented nuclei were considered as CAF-like cells (Figure 3). We detected comparable numbers of regular fibroblasts between normal and overweight/obese patients within the tumor and in tumor-adjacent adipose tissue (Table 4 and Figure 3). However, we found that fibroblasts with a CAF-like phenotype were significantly elevated in overweight/obese patients. In total, CAF-like cells were increased by 3.34-fold (*p* = 0.001), which mainly resulted from an increase in CAF-like cells within the tumor (3.31-fold; *p* = 0.002; Table 4). Furthermore, overweight/obese patients also had a higher ratio of CAF-like cells to regular fibroblasts (AT: *p* = 0.011, 2.72-fold; CT: *p* = 0.033, 1.94-fold; in total: *p* = 0.028, 1.8-fold; Table 4).

### 3.5. Changes in Adipocyte Morphology

**Figure 4:**
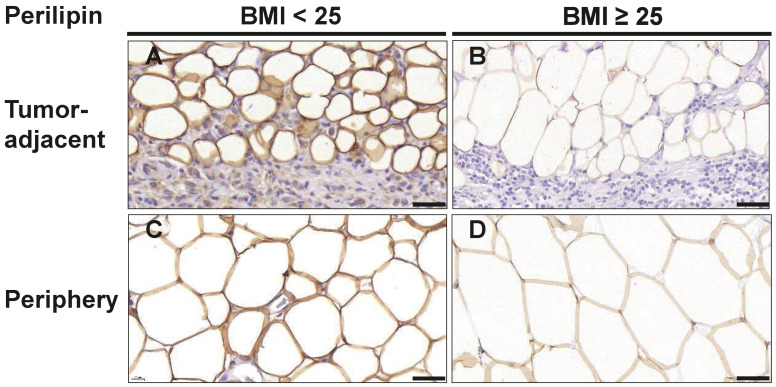
Adipocytes in breast tissue are larger in overweight/obese TNBC patients. Representative images of immunohistochemical staining for the adipocyte marker perilipin in tumor-adjacent adipose tissue (**A**,**B**) and adipose tissue ≥ 2 cm distant from the tumor margin (**C**,**D**) in normal weight (BMI_<25_) and overweight/obese patients (BMI_≥25_) with TNBC. Scale bar 50 µm.

Since we had observed several alterations in cellularity in the tumor-adjacent adipose tissue of overweight/obese TNBC patients, we next examined the morphology of adipocytes in more detail. Hence, we stained tissue sections with the adipocyte marker perilipin and determined the size of adipocytes directly adjacent to the invasive front of the tumor (1st row, 2nd row; Figure 4A,B) and at least 2 cm distant from the invasive front (Figure 4C,D). Of note, in overweight/obese patients, both tumor-adjacent and distant mammary adipocytes were larger than in normal-weight TNBC patients (Table 5, Figure 4).

Furthermore, the size of adipocytes increased with the distance to the tumor invasive front: Adipocytes directly adjacent to the tumor (1st row) had a median area of 1541 µm^2^ (IQR: 1043–2034) in samples of normal-weight patients as compared to 2377 µm^2^ (IQR: 1902–3350) in overweight/obese patients (*p* = 0.019). Adipocytes located in the second row adjacent to the tumor had a median area of 2345 µm^2^ (IQR: 1921–3018) in normal-weight patients as compared to 3849 µm^2^ (IQR: 2374–4623, *p* = 0.037) in overweight/obese patients. For adipocytes located at least 2 cm distant from the tumor invasive front, a median size of 5741 µm^2^ (IQR: 3720–6724) was observed in samples of the normal-weight group whereas a median size of 6910 µm^2^ (IQR: 5263–8176) was observed in the overweight/obese group. These observations are consistent with the notion that cancer-associated adipocytes at the invasive front may become delipidated and smaller through interaction with tumor cells.

### 3.6. Markers of Lipid Metabolism in TNBC Cells

**Figure 5:**
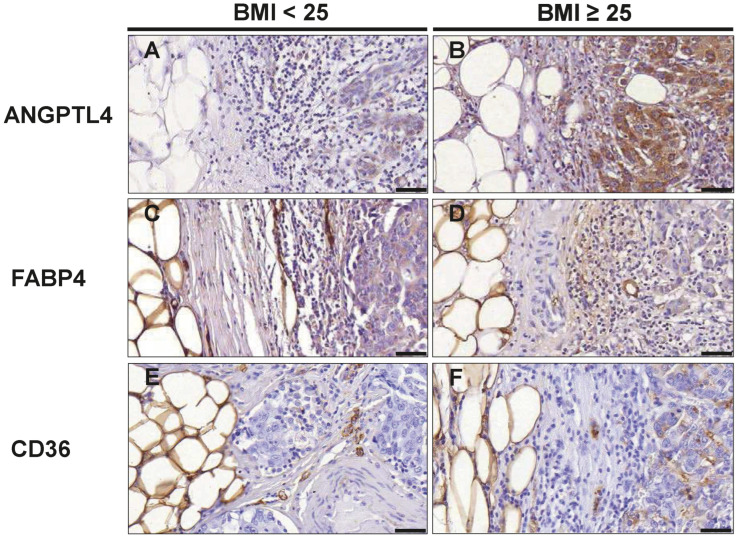
Evaluation of cancer cells at the tumor front for markers associated with fatty acid metabolism. Representative images of immunohistochemical staining with antibodies against ANGPTL4 (**A**,**B**); FABP4 (**C**,**D**) and CD36 (**E**,**F**) in normal weight (BMI_<25_) and overweight/obese patients (BMI_≥25_) with TNBC. Scale bar 50 µm.

Obesity itself as well as obesity-associated changes in the tumor microenvironment can induce re-programming of tumor cell metabolism towards lipid utilization. Particularly, adipocytes of the tumor microenvironment may serve as a rich source of fatty acids to fuel tumor cells. Thus, we investigated whether tumor cell expression at the invasive front of the proteins CD36, FABP4, and ANGPTL4, which are involved in the release, cellular uptake, and transport of fatty acids differs between normal and overweight/obese TNBC patients. CD36, FABP4, and ANGPTL4 were readily detectable in tumor cells by immunostaining (Figure 5). For a semiquantitative analysis, an immunoreactive score (IRS) was calculated for each marker by multiplying the staining intensity score with the percentage of the stained cells score. While the IRS for FABP4 was comparable between the normal and overweight/obese group (8.80 versus 9.07), the IRS for ANGTPL4 (6.00 versus 9.80; *p* = 0.026) and CD36 (2.15 versus 2.60; *p* = 0.041) were both significantly higher in marginal tumor cells in the overweight/obese group (Table 6).

## 4. Discussion

To date, the role of tumor-adjacent adipose tissue in TNBC, particularly in overweight/obese patients is not well understood. In this study, we focused on histologically characterizing the invasive tumor margin and adjacent mammary adipose tissue of the TME in normal-weight and overweight/obese patients with TNBC. We hypothesized that distinct histological changes occur in the adipose tissue TME in relation to BMI. Key findings include significantly larger adipocytes, increased numbers of CD163+ cells (M2-like macrophages), and decreased numbers of CD31+ and CD34+ cells (markers of angiogenesis) in the tumor-adjacent adipose tissue, as well as a higher frequency of CAF-like cells in overweight/obese TNBC patients. Additionally, the expression of ANGTPL4 and CD36, proteins involved in fatty acid metabolism, was increased in the marginal tumor cells of these patients.

Overweight/obese TNBC patients displayed larger mammary adipocytes, both in AT directly adjacent to cancer cells of the tumor front and in tumor-distant mammary fat. These results support the known association between adipocyte size and BMI [27,28], suggesting an increased hypertrophy of mammary adipocytes in overweight/obese patients with TNBC. Hypertrophic adipocytes are a hallmark of dysfunctional adipose tissue and associated with a pro-inflammatory secretome that may contribute to the progression and aggressiveness of breast cancer [7,29]. Indeed, mammary adipocyte hypertrophy was already identified as a negative prognostic factor in a cohort of pre- and post-menopausal women with mainly ER and PR positive breast cancer [30] and may thus also be of particular relevance in obese TNBC patients. Further, we observed that adipocytes directly adjacent to the invasive front were smaller than more distant ones, aligning with the concept of cancer-associated adipocytes (CAAs). CAAs are located in the TME and have undergone several molecular and phenotypic changes upon interaction with tumor cells. They are characterized by increased lipolysis, lower lipid content, smaller cell size, and elevated secretion of pro-inflammatory cytokines, which amplify local inflammation and the immune response [16,31].

Supporting an increased immune response, we found higher numbers of CD68+ and CD163+ (M2-like) macrophages in the tumor-adjacent adipose tissue of overweight/obese TNBC patients. This finding is consistent with the increased recruitment of macrophages to visceral and subcutaneous adipose tissue in obesity [9,32]. Generally, obesity has been associated with the promotion of a pro-inflammatory, antitumoral M1 adipose tissue macrophage phenotype in non-cancerous contexts [10,33]. Of note, the tumor-adjacent adipose tissue of obese patients showed a substantially larger increase of CD163+ macrophages (3.7-fold) than for CD68+ (1.7-fold) as compared to normal-weight TNBC patients. Further, CD163+ cells were more abundant than CD68+ cells, which was unexpected, as CD68 is typically recognized as a pan-macrophage marker, whereas CD163 is an established marker for immunosuppressive, protumoral M2-polarized macrophages [34]. A lower frequency of CD68+ macrophages has also been observed in studies of patients with liver metastasis from colorectal cancer [35] and melanoma patients [36]. While the exact underlying reasons remain unclear, this observation may be due to the limitations of IHC when using a single marker, as CD68 low-expressing macrophages, which have been reported in previous studies [37,38], could complicate detection. CD68 is predominantly expressed on the intracellular lysosomes and a weaker expression was identified in subpopulations such as immature macrophages [39,40,41]. It has also been shown that the sensitivity of staining for CD68 with different monoclonal antibodies depends on the pretreatment of tissue samples and varies by antibody [42]. Noteworthy, the PGM1 anti-CD68 monoclonal antibody, used in the present study, detects significantly fewer macrophages in IHC compared to the KP1 and EBM11 anti-CD68 monoclonal antibodies [42]. While CD68 and CD163 are widely used surrogate markers to investigate macrophage polarity and characterize tumor-associated macrophages, the binary classification of macrophages into M1 and M2 polarized activation states is overly simplistic [43]. In response to the diverse stimuli within the TME, macrophages exhibit remarkable plasticity, allowing them to adopt a variety of polarization states. This has been demonstrated in recent studies employing single-cell RNA sequencing, spatial transcriptomics, and metabolic profiling [43]. Interestingly, recent research has revealed significant functional and phenotypic heterogeneity among adipose tissue macrophage subpopulations [6], including an obesity-induced “metabolic activation” state that is phenotypically distinct from both M1 and M2 polarization [44]. Therefore, follow-up studies using more comprehensive molecular techniques are essential to gain a deeper understanding of macrophage subpopulations and their roles in the adipose tissue TME of obese TNBC patients.

Notwithstanding, our results on elevated CD163+ macrophages are in line with previous studies showing a positive correlation between CD163+ macrophages and hormone receptor negativity [45]. Moreover, it has been shown that tumor-associated macrophages (TAMs) mainly display an M2- phenotype [43], which has been linked to cancer progression, metastasis, and adverse survival outcomes in breast cancer patients [37,45,46,47]. Thus, the observed obesity-associated increase in CD163+ macrophages in the tumor-adjacent adipose tissue of TNBC patients may contribute to the worse prognosis of these patients. Given the immunosuppressive properties of CD163+ macrophages in the TME, partly due to their expression of immunosuppressive molecules such as programmed death-ligand 1 (PD-L1), targeted strategies to reprogram the immunosuppressive TME into a pro-inflammatory environment to enhance the efficacy of checkpoint inhibitor therapies may present a strategy to advance treatment options in overweight/obese TNBC patients [48].

Likewise, the density of crown-like structures (CLSs) in breast adipose tissue has been associated with a worse prognosis in breast cancer patients [6,10,11,49]. CLSs consist of macrophage clusters surrounding dying adipocytes and are a marker of adipose tissue inflammation [50]. We observed significantly more CLSs in tumor-adjacent AT in the overweight/obese group, corroborating the theory that adiposity is strongly associated with the prevalence of CLSs in mammary adipose tissue [6,10,11,49,51].

Obesity may also affect other cell types (e.g., endothelial cells and fibroblasts) and physiological processes (e.g., matrix synthesis and angiogenesis) within the adipose tissue microenvironment in cancer [6]. In line with this, we observed significantly lower numbers of CD31+ and CD34+ cells, surrogate markers for endothelial cells and microvessel formation, in the tumor-adjacent adipose tissue of overweight/obese TNBC patients, suggesting that adiposity-related changes in the breast adipose tissue impact vascularization. Furthermore, we identified substantial differences in fibroblast populations between normal-weight and overweight/obese TNBC patients. While the abundance of regular fibroblasts was comparable between the groups, the number of CAF-like cells was significantly higher in both the adipose and cancer tissue of overweight/obese TNBC patients. In our study, CAF-like cells were distinguished from regular fibroblasts in vimentin stains, based on their morphology. Although they share a spindle shape, CAFs are larger, possess multiple branches of cytoplasm, and have indented nuclei under light microscopy [52]. Since we did not further characterize these cells by staining for specific CAF marker proteins—such as the fibroblast activation protein α (FAPα) and α-smooth muscle actin (αSMA) [12] —we refer to them as CAF-like cells to account for this limitation. In general, CAFs are hyper-activated fibroblasts and are among the most abundant stromal components in TME. They exhibit significant heterogeneity [53] and play crucial roles in various aspects of cancer pathogenesis, including matrix remodeling and the production of growth factors, cytokines, and chemokines that contribute to the M2 polarization of TAMs and tumor cell invasion [12,54]. Our finding of increased CAF-like cells in overweight/obese TNBC patients is consistent with the elevated number of CD163+ M2-macrophages in tumor-adjacent adipose tissue observed in these patients.

Obesity and more aggressive breast cancer cells are often characterized by alterations in lipid and fatty acid metabolism. The metabolic rewiring of tumor cells towards lipid utilization is increasingly recognized as a hallmark of cancer aggressiveness and progression [55]. Recent investigations have provided evidence of FABP4, CD36, and ANGPTL4, proteins involved in the release, uptake, and intracellular handling of triglycerides and fatty acids, in breast cancer pathology [17,21,22,23]. While FABP4 expression was comparable between the groups, we observed higher expression levels of CD36 and ANGPTL4 in tumor cells at the invasive front in overweight/obese TNBC patients. CD36 is a fatty acid transport protein, which has been shown to facilitate the uptake of fatty acids released from adipocytes into cancer cells [56]. While the expression of CD36 has not been extensively studied in the context of obesity and TNBC, elevated CD36 expression has been reported to promote cancer cell survival in HER2-positive breast cancer and to initiate metastasis formation [57,58]. Similarly, ANGPTL4 has been shown to enhance breast cancer cell invasion and metastasis to the lung [59,60]. In previous work, our group demonstrated that upregulated ANGPTL4 expression in triple-negative MDA-MB-231 cells, upon co-culture with adipose tissue-conditioned media, increases proliferation, motility, and invasiveness in vitro [17]. The increased expression of ANGPTL4 observed in tumor cells at the invasive front in the overweight/obese group now provides novel in vivo evidence supporting a role for ANGPTL4 in obesity-related breast cancer. Collectively, the elevated expression of CD36 and ANGPTL4 in overweight/obese TNBC patients may suggest more extensive metabolic reprogramming of cancer cells in response to the obese adipose tissue tumor microenvironment.

A limitation of the present investigation is the small sample size of the study cohort. However, the cohort is well characterized, consisting solely of patients with triple-negative breast cancer, and the subjects in the normal and overweight/obese groups were carefully matched. Despite the limited statistical power, we identified several significant differences in the adipose tissue microenvironment in this pilot study. These findings warrant follow-up investigations in larger, independent cohorts to validate the results.

## 5. Conclusions

Our study provides evidence for BMI-related changes in the adipose tissue tumor microenvironment of TNBC patients. These changes encompass the polarization state and frequency of macrophages, crown-like structures as markers of adipose tissue inflammation, the abundance of CAF-like cells, and the size of mammary adipocytes in the direct tumor vicinity. Furthermore, overweight/obesity was associated with increased expression of CD36 and ANGPTL4 in tumor cells at the invasive front, suggesting altered lipid metabolism and metabolic reprogramming in the tumor cells of these patients. Further studies are needed to determine whether these changes in the adipose tissue tumor microenvironment are causally linked to the more aggressive tumor phenotype and worse outcomes observed in obese breast cancer patients.

## Figures and Tables

**Table 1:** Clinicopathologic characteristics of the study patients.

	Group 1: BMI < 25 (n = 10)	Group 2: BMI ≥ 25 (n = 20)	*p*-Value
Age (years)			
Median (IQR)	61.00 (52.75–78.25)	65.00 (56.00–79.50)	0.991
BMI			
Median (IQR)	22.40 (20.93–23.25)	29.30 (26.30–33.50)	<0.001
Histological Grade			
I	0	0	
II	3	3	
III	7	17	
Tumor Stage			
T1	6	12	
T2	4	6	
T3	0	2	
Nodal Metastasis [%]	40.00	20.00	
DCIS [%]	70	40	
Mitosis-Score			
1	1	0	
2	3	7	
3	6	13	

**Table 2:** Distribution of CD68+ and CD163+ macrophages and CLS.

	BMI < 25	BMI ≥ 25	*p*-Value
CD68			
Adipose Tissue	1.72 (0.40–2.48)	2.90 (1.55–4.30)	0.038
Cancer Tissue	7.62 (1.15–15.10)	14.00 (9.55–29.00)	0.119
Total	9.33 (1.48–17.58)	16.70 (12.50–33.00)	0.091
CD163			
Adipose Tissue	2.80 (2.19–7.50)	10.45 (4.98–21.10)	0.011
Cancer Tissue	9.50 (8.58–32.75)	36.14 (24.55–40.40)	0.062
Total	11.50 (10.75–40.25)	46.65 (32.58–54.98)	0.039
CLS	0.25 (0.00–2.08)	1.70 (0.85–3.30)	0.001

**Table 3:** Distribution of CD31+ and CD34+ cells.

	BMI < 25	BMI ≥ 25	*p*-Value
CD31			
Adipose Tissue	4.20 (3.75–6.31)	2.40 (1.50–4.40)	0.018
Cancer Tissue	9.75 (7.40–12.41)	10.80 (8.80–13.40)	0.453
Total	14.75 (11.35–20.25)	13.60 (11.10–17.10)	0.713
CD34			
Adipose Tissue	14.60 (5.80–31.50)	5.20 (2.23–10.10)	0.045
Cancer Tissue	27.50 (14.70–59.70)	18.20 (10.35–40.76)	0.275
Total	42.10 (18.90–91.90)	23.20 (12.80–51.15)	0.211

**Table 4:** Distribution of fibroblasts and CAF-like cells.

	BMI < 25	BMI > 25	*p*-Value
Vimentin			
CAF-like cells			
Adipose Tissue	2.10 (1.19–3.95)	4.75 (1.90–8.38)	0.053
Cancer Tissue	6.00 (5.16–13.20)	19.85 (10.95–37.20)	0.002
Total	7.60 (7.03–18.75)	25.39 (12.45–44.89)	0.001
Fibroblasts			
Adipose Tissue	7.13 (3.81–9.75)	5.68 (4.50–8.78)	0.660
Cancer Tissue	23.30 (17.04–27.70)	23.50 (15.61–25.98)	0.864
Total	29.30 (22.20–37.45)	29.75 (21.19–36.78)	0.829
CAFs/Fibroblasts			
Adipose Tissue	0.36 (0.19–0.56)	0.98 (0.41–1.48)	0.011
Cancer Tissue	0.34 (0.26–0.68)	0.66 (0.49–1.75)	0.033
Total	0.35 (0.26–0.65)	0.63 (0.52–1.80)	0.028

**Table 5:** Distribution of adipocyte size (perilipin staining) in different tissue areas among patient groups.

	BMI < 25	BMI ≥ 25	*p*-Value
Perilipin			
Avg. adipocyte area (µm^2^)			
1st Row	1541 (1043–2034)	2377 (1902–3350)	0.019
2nd Row	2345 (1921–3018)	3849 (2374–4623)	0.037
Periphery (2 cm distance to tumor)	5741 (3720–6724)	6910 (5263–8176)	0.067

**Table 6:** ANGPTL4, FABP4 and CD36 immunoreactivity scores.

	BMI < 25	BMI ≥ 25	*p*-Value
ANGPTL4			
Score	6.00 (4.64–7.90)	9.80 (6.58–10.60)	0.026
FABP4			
Score	8.80 (6.35–10.80)	9.07 (4.38–10.93)	0.949
CD36			
Score	2.15 (1.88–2.45)	2.60 (2.40–3.20)	0.041

## Data Availability

The data that support the findings of this study are available from the authors upon reasonable request.

## Supplementary Materials

The following supporting information can be downloaded at: https://www.mdpi.com/article/10.3390/cancers16203515/s1, Table S1: Antibodies and dilutions.
