# Supplementary material for: Histologic Characterization of Tumor-Adjacent Mammary Adipose Tissue in Normal-Weight and Overweight/Obese Patients with Triple-Negative Breast Cancer
Source: Cancers (Basel). 2024 Oct 17;16(20):3515. doi: 10.3390/cancers16203515 (PMC11506523; doi:10.3390/cancers16203515)
Supplement: Supplementary file 1 [file cancers-16-03515-s001.zip › cancers-3216569-supplementary.pdf]

cancers MDPI

Histologic Characterization of Tumor-Adjacent Mammary Adipose Tissue in Normal-Weight and Overweight/Obese Patients with Triple-Negative Breast Cancer

Table S1. Antibodies and dilutions.

	Antibody	Company	Catalogue number	Dilution
**Manual staining**	Anti-Perilipin goat IgG poly	Abcam	ab61682	1:200
	Anti-ANGPTL4 rabbit IgG poly	Proteintech	18374-1-AP	1:250
	Anti-FABP4 rabbit IgG poly	Sigma Aldrich	HPA002188	1:200
	Anti-CD36 rabbit IgG mono	Cell Signaling	14347	1:200
	Isotype rabbit IgG	Thermo Fischer	02-6102	-
	Isotype goat IgG	Thermo Fischer	02-6202	-
**Automated staining**	Anti-CD31 mouse IgG mono	Dako	M0823	1:40
	Anti-CD34 mouse IgG mono	Dako	M7165	1:150
	Anti-CD68 PG-M1 mouse IgG mono	Dako	M0876	1:200
	Anti-CD163 mouse IgG mono	Leica	NCL-CD163	1:100
	Anti-Vimentin mouse IgG mono	Leica	NCL-L-VIM-V9	1:750
**Secondary antibodies (Perilipin, ANGPTL4, FABP4, CD36)**	Histofine Simple Stain MAX PO (Multi)	Nichirei Biosciences	414154F	-
	Histofine Simple Stain MAX PO (Goat)	Nichirei Biosciences	414161F	-
